# Validating genetic risk associations for ovarian cancer through the international Ovarian Cancer Association Consortium

**DOI:** 10.1038/sj.bjc.6604820

**Published:** 2009-01-06

**Authors:** C L Pearce, A M Near, D J Van Den Berg, S J Ramus, A Gentry-Maharaj, U Menon, S A Gayther, A R Anderson, C K Edlund, A H Wu, X Chen, J Beesley, P M Webb, S K Holt, C Chen, J A Doherty, M A Rossing, A S Whittemore, V McGuire, R A DiCioccio, M T Goodman, G Lurie, M E Carney, L R Wilkens, R B Ness, K B Moysich, R Edwards, E Jennison, S K Kjaer, E Hogdall, C K Hogdall, E L Goode, T A Sellers, R A Vierkant, J C Cunningham, J M Schildkraut, A Berchuck, P G Moorman, E S Iversen, D W Cramer, K L Terry, A F Vitonis, L Titus-Ernstoff, H Song, P D P Pharoah, A B Spurdle, H Anton-Culver, A Ziogas, W Brewster, V Galitovskiy, G Chenevix-Trench, Australian Cancer Study (Ovarian Cancer), Australian Ovarian Cancer Study Group,

**Affiliations:** 1 Department of Preventive Medicine, Keck School of Medicine, University of Southern California, Los Angeles, CA, 90033, USA; 2 Department of Epidemiology, University of Michigan School of Public Health, Ann Arbor, Michigan, 48105, USA; 3 Keck School of Medicine, University of Southern California, Los Angeles, CA, 90033, USA; 4 Gynaecological Cancer Research Laboratories, UCL EGA Institute for Women's Health, University College London, London, WC1E 6DD, UK; 5 UCL EGA Institute for Women's Health, Gynaecological Cancer Research Centre, London, W1T 7DN, UK; 6 The Queensland Institute of Medical Research, Post Office Royal Brisbane Hospital, Herston, Brisbane, QLD 4029, Australia; 7 Epidemiology Program, Division of Public Health Sciences, Fred Hutchinson Cancer Research Center, Seattle, WA 98109-1024, USA; 8 Department of Health Research and Policy, Division of Epidemiology, Stanford University School of Medicine, Stanford, CA 94305, USA; 9 Department of Cancer Genetics, Roswell Park Cancer Institute, Buffalo, NY 14263, USA; 10 Cancer Research Center, University of Hawaii, Honolulu, Hawaii, 96813, USA; 11 The University of Texas School of Public Health, Houston, TX 77030, USA; 12 Department of Cancer Prevention and Control, Roswell Park Cancer Institute, Buffalo, NY, 14263, USA; 13 Department of OB/GYN/RS, Magee-Women's Hospital, University of Pittsburgh Medical Center, Pittsburgh, PA 15213, USA; 14 Gynecologic Oncologists of Northeast Ohio, Akron, OH 44302, USA; 15 Department of Virus, Hormones and Cancer, Institute of Cancer Epidemiology, Danish Cancer Society, 2100 Copenhagen, Denmark; 16 Gynaecologic Clinic, Juliane Marie Centre, Rigshospitalet, University of Copenhagen, 2100 Copenhagen, Denmark; 17 Mayo Clinic College of Medicine, Rochester, MN, 55905, USA; 18 H. Lee Moffitt Cancer Center and Research Institute, MRC CANCONT, Tampa, FL, 33612, USA; 19 Cancer Prevention and Control Research Program, Duke University Medical Center, Durham, NC 27710, USA; 20 Division of Gynecologic Oncology, Duke University Medical Center, Durham, NC, 27710, USA;; 21 Department of Statistical Sciences, Duke University, Durham, NC 27708-0251, USA; 22 Brigham and Women's Hospital, Boston, MA 02115, USA; 23 Department of Community and Family Medicine and of Pediatrics, Dartmouth Medical School, Lebanon, NH 03756, USA; 24 Strangeways Research Laboratory, Department of Oncology, University of Cambridge, Cambridge, CB1 8RN, UK; 25 Department of Epidemiology, School of Medicine, University of California, Irvine, CA 92697, USA; 26 Department of OB/GYN, UCI Medical Center, Chao Clinical Cancer Center, Orange, CA 92868, USA; 27 Peter MacCallum Cancer Centre, East Melbourne, VIC 3002, Australia

**Keywords:** ovarian cancer, genetic susceptibility, oestrogen metabolism, CYP3A4, pooled-analyses

## Abstract

The search for genetic variants associated with ovarian cancer risk has focused on pathways including sex steroid hormones, DNA repair, and cell cycle control. The Ovarian Cancer Association Consortium (OCAC) identified 10 single-nucleotide polymorphisms (SNPs) in genes in these pathways, which had been genotyped by Consortium members and a pooled analysis of these data was conducted. Three of the 10 SNPs showed evidence of an association with ovarian cancer at *P*⩽0.10 in a log-additive model: rs2740574 in CYP3A4 (*P*=0.011), rs1805386 in LIG4 (*P*=0.007), and rs3218536 in XRCC2 (*P*=0.095). Additional genotyping in other OCAC studies was undertaken and only the variant in CYP3A4, rs2740574, continued to show an association in the replication data among homozygous carriers: OR_homozygous(hom)_=2.50 (95% CI 0.54-11.57, *P*=0.24) with 1406 cases and 2827 controls. Overall, in the combined data the odds ratio was 2.81 among carriers of two copies of the minor allele (95% CI 1.20–6.56, *P*=0.017, p_het_ across studies=0.42) with 1969 cases and 3491 controls. There was no association among heterozygous carriers. CYP3A4 encodes a key enzyme in oestrogen metabolism and our finding between rs2740574 and risk of ovarian cancer suggests that this pathway may be involved in ovarian carcinogenesis. Additional follow-up is warranted.

Ovarian cancer is the second most common gynaecologic cancer and the leading cause of gynaecologic cancer death; it is estimated that there were ∼22 000 new diagnoses of ovarian cancer and ∼15 000 deaths from the disease in the United States in 2007. In some families Mendelian inheritance of ovarian cancer is observed and high penetrance alleles have been identified in several genes including BRCA1, BRCA2, and DNA mismatch repair genes. However, disease-associated alleles of these genes account for less than 40% of the inherited variance in disease risk (Antoniou and Easton, 2006). Population-based case–control studies have described a two to three-fold increased risk in first-degree relatives of ovarian cancer patients (Godard *et al*, 1998; Stratton *et al*, 1998) and heritability is estimated to be approximately 22% (Lichtenstein *et al*, 2000). Ovarian cancer is probably a complex genetic disease with locus and allelic heterogeneity (Lander and Schork, 1994), and such complex traits are likely to be because of combinations of common, less penetrant alleles (Risch and Merikangas, 1996). However, the common alleles that might be responsible for susceptibility to ovarian cancer remain poorly understood.

The most powerful approach for identifying common, low-penetrance disease susceptibility alleles is the genetic association study. Until recently, most association studies have focused on the candidate gene approach in which common genetic variation in biologically relevant pathways is tested for association with disease. However, this approach has had limited success, and few initial findings of positive associations have been replicated in subsequent studies (Ioannidis *et al*, 2001; Lohmueller *et al*, 2003). One of the main reasons for these failures has been the limited power of small studies to detect associations at highly stringent levels of statistical significance. This is a particular problem in ovarian cancer where the largest individual studies have fewer than 2000 cases.

The Ovarian Cancer Association Consortium (OCAC) was formed in 2005 to provide a forum for testing candidate ovarian cancer susceptibility alleles in multiple studies with a large combined sample size (Gayther *et al*, 2007; Pearce *et al*, 2008; Ramus *et al*, 2008). Prior to the formation of the OCAC most groups had been carrying out candidate gene studies autonomously, although in many instances they had been studying the same candidate single nucleotide polymorphisms (SNPs) and genes. The purpose of the current study was to evaluate the evidence for association in SNPs, which had already been genotyped by multiple studies by combining the existing data. Where evidence for association emerged from these data, the associated SNPs were then genotyped in additional OCAC studies for replication.

## Materials and methods

### Study populations

The Ovarian Cancer Association Consortium is comprised of ovarian cancer case–control and cohort studies conducted around the world (Gayther *et al*, 2007; Pearce *et al*, 2008; Ramus *et al*, 2008). Included in this report are data from 16 OCAC studies. Ten of these are from the USA: the Diseases of the Ovary and their Evaluation (DOVE), the Genetic Epidemiology of Ovarian Cancer Study (GEOCS; previously FROCS) (Auranen *et al*, 2005; Song *et al*, 2006), the Hawaii Ovarian Cancer Study (HAWAII) (Goodman *et al*, 2001b), the Hormones and Ovarian Cancer Prediction Study (HOPE) (Pearce *et al*, 2008a), the Mayo Clinic Ovarian Cancer Case–Control Study (MAYO) (Sellers *et al*, 2005), the North Carolina Ovarian Cancer Study (NCOCS) (Berchuck *et al*, 2004), the New England-based Case–Control Study (Terry *et al*, 2005), the Ovarian Contraceptive and Reproductive Experiences study (Holt *et al*, 2007), the Orange and San Diego Counties, California (UCI) study and the USC/Los Angeles County Case–Control Studies of Ovarian Cancer (USC) (Pearce *et al*, 2008b). There are data from three European studies: the Danish Malignant Ovarian Cancer Study (MALOVA) (Auranen *et al*, 2005; Song *et al*, 2006), the UK SEARCH Ovarian Cancer Study (SEARCH) (Auranen *et al*, 2005; Song *et al*, 2006), and the UK Ovarian Population Study (UKOPS) (Ramus *et al*, 2008). Finally, data were contributed by three studies from Australia: the Australian Cancer Study (ACS) (Merritt *et al*, 2008), the Australian Ovarian Cancer Study (AOCS) (Merritt *et al*, 2008), and Survey of Women's Health (SWH) (Spurdle *et al*, 2000). ACS and AOCS were combined for analysis purposes. Details of all but DOVE and UCI have been published earlier.

All studies have Institutional Review Board/Human Ethics Committee approval for the work presented. In addition, Duke University and the University of Southern California have Institutional Review Board approval as data coordinating centers.

### SNP selection

At the inception of the OCAC and annually thereafter, members have provided a list of SNPs genotyped in their study population. By early 2007, there were 10 SNPs which had been genotyped by at least three groups: rs4680 in COMT, rs4646903 and rs1048943 in CYP1A1, rs1056836 in CYP1B1, rs2740574 in CYP3A4, rs743572 in CYP17, rs3020450 in ESR2, rs1805386 in LIG4, rs3218536 in XRCC2 and rs861539 in XRCC3. Some of these data have earlier been published by individual groups (Spurdle *et al*, 2000, 2002; Goodman *et al*, 2001a, 2001b; Garner *et al*, 2002; Terry *et al*, 2003; Auranen *et al*, 2005; Sellers *et al*, 2005; Webb *et al*, 2005; Beesley *et al*, 2007; Holt *et al*, 2007; Pearce *et al*, 2008b). The original data on these 10 SNPs were then submitted to a central database for combined analysis. SNPs that showed some evidence for association in original data were also genotyped in additional OCAC studies for replication purposes.

### Genotyping and quality control

The original data had been genotyped using a variety of methods described in the reports of these individual data (see Table 1 for references). For the replication genotyping, the 5′ nuclease Taqman allelic discrimination assay (Taqman; Applied Biosystems, Foster City, CA, USA) was used by DOVE, HAWAII, HOPE, MALOVA, MAYO, SEARCH, UCI, UKOPS and USC using centrally supplied probes. ACS and AOCS used the Sequenom iPlex gold genotyping platform (Sequenom Inc., San Diego, CA, USA).

For all genotype data, genotype frequencies by ethnic group and study in controls were tested for deviation from Hardy–Weinberg equilibrium (HWE). For the replication genotyping effort, we applied the OCAC quality control guidelines which stipulated intermixing of cases and controls on genotyping plates, including duplicate samples with no less than 98% concordance rate, overall call rate by each study of 95% or greater and call rate for each plate of at least 90%. In addition, consistency across labs was confirmed by genotyping a common set of 95 DNAs (90 CEPH trios and five duplicate samples; HAPMAPPT01 provided by Coriell, Camden, NJ, USA) with the requirement of >98% concordance in genotype calls.

### Data analysis

Each group provided age and race/ethnicity for all participants and tumour histology for cases. Data analyses were restricted to invasive epithelial ovarian cancers and included White, Black and Latina individuals. Unconditional logistic regression stratified on study, age (five groups: <40, 40–49, 50–59, 60–69, 70+ years), and race/ethnicity, was used to analyze the data (SAS Version 9.1, Cary, NC, USA). Each SNP was evaluated under three genetic models: a co-dominant log additive model, a dominant model and a recessive model. Results are given for the heterozygous and homozygous risk estimates.

Information on a first-degree family history of ovarian cancer was available on approximately 75% of cases and controls. Stratified analyses were conducted on this subset of subjects.

The original and replication data were analyzed separately and combined using the same methods. Heterogeneity across studies was tested using the likelihood ratio test by fitting models with and without interaction terms for study and SNP based on the final model presented in the results.

Published data from non-OCAC members were available on three SNPs: rs4680 (COMT) (Goodman *et al*, 2000), rs4646903 (CYP1A1, also known as CYP1A1 Msp1) (Sugawara *et al*, 2003) and rs1048943 (CYP1A1, also known as CYP1A1^*^3) (Aktas *et al*, 2002; Sugawara *et al*, 2003). The odds ratios and 95% confidence intervals from these published reports were abstracted and a meta-analysis was conducted utilizing these data as well as the OCAC data using Stata (Version 9, StataCorp, College Station, TX, USA). Both random and fixed-effects models were evaluated.

## Results

Details of the 16 case–control studies that provided data for these analyses are shown in Table 1. Genotype frequencies in White controls were consistent with HWE for all SNPs/studies except rs2740574 (CYP3A4) in HAW (*P*=0.04) (Supplementary Table 1). These data were retained in the analyses as the deviation was small, the clustering was unambiguous and given the number of SNP/study combinations evaluated it is likely to represent a chance finding.

Seven of the 10 SNPs showed no evidence of an association with invasive epithelial ovarian cancer (ovarian cancer; Table 2). These SNPs were not further evaluated in the OCAC, but three of them (COMT rs4680, CYP1A1 rs4646903, and CYP1A1 1048943) had been studied by groups outside of the OCAC and thus meta-analyses of the published literature and OCAC data were conducted. Neither the COMT variant nor the CYP1A1 rs4646903 variant were statistically significantly associated with ovarian cancer risk on meta-analysis (data not shown).

The CYP1A1 rs1048943 which showed a non-statistically significant 10% increased risk of ovarian cancer per copy of the minor allele carried in the OCAC dataset (95% CI 0.77–1.57) was statistically significantly associated with risk of ovarian cancer on meta-analysis with the published literature and the OCAC data (OR_additive(add)_=1.81, 95% CI 1.36–2.40, *P*<0.001) under a fixed effects model, but not a random-effects model (OR_add_=1.61, 95% CI 0.74–3.51, *P*=0.23). The meta-analysis also revealed significant heterogeneity across studies (*P*<0.001).

**Figure 1:**
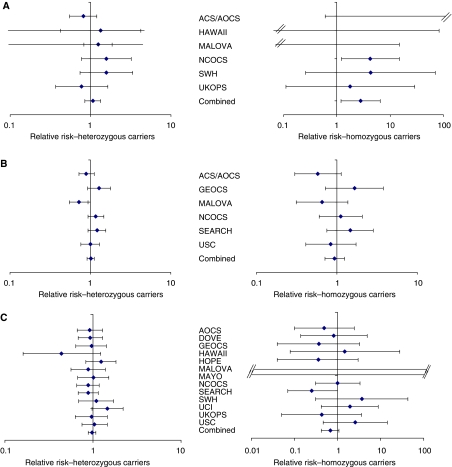
Forest plots of the study-specific and summary-relative risk and 95% confidence intervals for the association between ovarian cancer risk and three SNPs in the CYP3A4, LIG4 and XRCC2 genes. (**A**) rs2740574 in CYP3A4. The overall summary ovarian cancer risk among heterozygous carriers is 1.07 (0.85–1.34, *P*=0.57) and homozygous carriers is 2.81 (95% CI 1.20–6.56, *P*=0.017). (**B**) rs1805386 in LIG4. The overall summary ovarian cancer relative risk among heterozygous carriers is 1.01 (95% CI 0.91–1.12, *P*=0.82) and homozygous carriers is 0.94 (95% CI 0.71–1.25, *P*=0.68). (**C**) rs3218536 in XRCC2. The overall summary ovarian cancer relative risk among heterozygous carriers is 0.97 (95% CI 0.88–1.08, *P*=0.61) and homozygous carriers 0.68 (95% CI 0.42–1.09, *P*=0.11).

Three SNPs showed evidence of an association with ovarian cancer in the original OCAC data and were evaluated in additional case–control studies from OCAC members (Table 3; Figure 1). Based on original genotype data (three studies: 563 cases, 664 controls), CYP3A4 rs2740574 was associated with an increased risk of invasive ovarian cancer among women who carried two copies of the minor allele (*P*=0.015). There was a 3.7-fold increased risk associated with carrying two copies of the minor allele. Similar results were found when this SNP was genotyped by three additional OCAC sites in an additional 1406 cases and 2827 controls, but the confidence interval was wide (OR_homozygous(hom)_=2.50, 95% CI 0.54–11.54, *P*=0.24; Table 3) because the follow-up data consisted of White individuals in which the minor allele frequency was very low. In the combined dataset, women carrying two copies of this allele had a 2.8-fold increased risk of ovarian cancer (OR_hom_=2.81, 95% CI 1.20–6.56, p_hom_=0.017; Table 3; Figure 1). There was no evidence of heterogeneity across study sites (p_het_=0.42). This finding was consistent across histological subtypes (Table 4). There was no evidence of statistical interaction with family history (*P*=0.52).

The minor allele frequency for rs2740574 in White controls was 3.5%, compared with 59.7% in Blacks. The association between this CYP3A4 variant and risk of ovarian cancer was consistent across these two racial/ethnic groups in the combined existing and follow-up data: OR_hom_ in Blacks=6.82 (95% CI 0.73–63.86, *P*=0.093) and OR_hom_ in Whites=2.40 (95% CI 0.67–8.68, *P*=0.18).

There was a 21% increased risk of ovarian cancer associated with the rs1805386 variant in LIG4 among heterozygous carriers and a 34% increased risk among homozygous carriers (*P*=0.013 and *P*=0.15, respectively; Table 3) in the original data. When this SNP was genotyped by four additional OCAC sites (1691 cases and 2944 controls) a decreased risk of ovarian cancer was observed (OR_heterozygous (het)_=0.87, *P*=0.046 and OR_hom_=0.67, *P*=0.056; Table 3). After combining the original and replication data there was no association between rs1805386 and risk of ovarian cancer (OR_het_=0.97, *P*=0.61, OR_hom_=0.68, *P*=0.11; Table 3; Figure 1) in 3321 cases and 5140 controls. There was a significant heterogeneity across the study sites (*P*=0.001); the heterogeneity was not attributable to any single study. There was no evidence of statistical interaction with family history (*P*=0.50).

We found borderline evidence of a decrease in ovarian cancer risk associated with the XRCC2 variant rs3218536 using a log-additive model in the original data provided by six OCAC sites (OR=0.89, 95% CI 0.78–1.02, *P*=0.095) with 2763 cases and 5479 controls. This SNP was genotyped by seven additional OCAC sites comprising 2551 cases and 4005 controls and no association was observed with ovarian cancer risk (Table 3). In the combined data there was no association between rs3218536 and risk of ovarian cancer (Table 3; Figure 1). There was neither any evidence of heterogeneity across study sites (*P*=0.30), nor histological subtype-specific effects. There was also no evidence of statistical interaction with family history (*P*=0.25).

## Discussion

We evaluated the association between 10 SNPs from nine genes and ovarian cancer risk using existing data from 16 studies that participate in the OCAC. We found borderline evidence for association with ovarian cancer risk for three of these variants and replication genotyping was carried out to provide more definitive evidence of association. In the combined data, only rs2740574 in CYP3A4 was associated with disease among women who carried two copies of the minor allele (*P*=0.017). This finding was consistent across the original and replication data and also across White and Black racial/ethnic groups and histological subtypes (Table 4).

CYP3A4 encodes an enzyme critical for oxidation of oestrogens (Keshava *et al*, 2004) and its inhibition results in higher circulating oestrogen levels (Monroe *et al*, 2007). Given that exposure to oestrogen is associated with the risk of ovarian cancer (Beral *et al*, 2007) it is plausible that the CYP3A4 rs2740574 variant might influence ovarian cancer development through decreased expression of the gene and thus reduced metabolism of oestrogen. In addition to hormone metabolism, CYP3A4 is also involved in the metabolism of approximately half of all marketed drugs, therefore individuals with differences in CYP3A4 expression might have different responses to any number of exogenous compounds, including oral contraceptives, an ovarian cancer protective factor.

The rs2740574 SNP is in the promoter region of the gene, but efforts to show a functional effect of this variant have proved difficult. Spurdle *et al* (2002) evaluated multiple reporter gene constructs, but found no differences in the transcription between the putative disease and wild-type allele; the results of other functional studies have been equivocal (Ball *et al*, 1999; Westlind *et al*, 1999; Amirimani *et al*, 2003; Rodriguez-Antona *et al*, 2005). Alternatively, it may be that rs2740574 is simply in linkage disequilibrium with the causal variant, which remains to be identified. Using data from the International Haplotype Map Project (HapMap) to examine the region that includes CYP3A4, CYP3A5 and CYP3A7 genes and the 20 kb flanking this region, there are six variants in perfect linkage disequilibrium with rs2740574. Five of these variants are in the region immediately 5′ of CYP3A4 and one is in intron 2 of the gene. Any of these SNPs, or an unidentified variant in linkage disequilibrium, could be the causal allele. At present, there are no data to support a functional role for any of these variants in ovarian cancer development. Additional follow-up is warranted to confirm the association between this CYP3A4 variant and risk of ovarian cancer, and ideally these studies should be performed in different racial groups.

Two additional variants, one in LIG4 and the other in XRCC2, evaluated as part of this project, showed association in the original data provided by OCAC sites, but no association was observed after replication genotyping was carried out in additional OCAC studies. The LIG4 variant, rs1805386, showed significant heterogeneity across studies (*P*=0.001) for reasons that could not be elucidated. It is perplexing that the original data suggested a statistically significant positive association with risk, whereas the replication data suggested an inverse relationship. In the absence of any reasonable explanation it is likely that the heterogeneity was due to chance. The failure to replicate the association with the XRCC2 variant rs3218536 was not surprising as the evidence of association in the original data was weak under a co-dominant genetic model (*P*=0.095).

Seven additional variants did not show an association with the risk of ovarian cancer in the OCAC dataset. Three of these variants have been studied by groups outside the OCAC: rs4680 (COMT) (Goodman *et al*, 2000), rs4646903 (CYP1A1, also known as CYP1A1 Msp1) (Sugawara *et al*, 2003) and rs1048943 (CYP1A1, also known as CYP1A1^*^3) (Aktas *et al*, 2002; Sugawara *et al*, 2003). Neither the COMT variant nor the CYP1A1 rs4646903 variant were associated with the risk of ovarian cancer on meta-analyses of the published literature and the OCAC data.

The CYP1A1 rs1048943 variant was associated with a non-statistically significant increased risk of ovarian cancer per copy of the minor allele carried in the OCAC dataset and in a Japanese population (OR_add_=1.16, 95% CI 0.44-3.05) (Sugawara *et al*, 2003); in a Turkish population it was associated with a statistically significant increased risk of ovarian cancer (OR_add_=6.20, 95% CI 3.62–10.46) (Aktas *et al*, 2002). Overall, the meta-analysis of the published literature and the OCAC data showed a statistically significant increased risk of ovarian cancer (OR_add_=1.81, 95% CI 1.36–2.40, *P*<0.001) under a fixed-effects model, but not a random-effects model (OR_add_=1.61, 95% CI 0.74–3.51, *P*=0.23). The meta-analysis also revealed significant heterogeneity across studies (*P*<0.001) bringing this association into question. Further follow-up of this variant may be warranted.

There are several limitations to this study. The association with CYP3A4 rs2740574 was consistent in the original and replication data and was consistent across racial/ethnic groups, but it was restricted to homozygous carriers of the minor allele. The risk allele is rare in Whites and the result is based on few cases and while the SNP is common in Blacks there are few included in this study. Also, four of the SNPs (rs4646903 and rs1048943 in CYP1A1, rs1056836 in CYP1B1, and rs743572 in CYP17), which were not statistically significantly associated with ovarian cancer and therefore not followed up by the OCAC, were associated with ∼10% increased or decreased risk of ovarian cancer per copy of the allele carried. This size of effect is of the same magnitude as those observed in a recent breast cancer genome-wide association scan (Easton *et al*, 2007). Some of these results could be false negatives as we were only powered to detect odds ratios of ∼1.18 and higher for these SNPs with 80% power and an α of 0.05. We also observed significant heterogeneity with the SNP in LIG4, which may have been a chance finding, but may have resulted from underlying differences in the study populations, an issue with pooled-analyses.

In conclusion, we have identified a possible association between an SNP (rs2740574) in the key oestrogen-metabolizing gene CYP3A4 and ovarian cancer risk. Follow-up of this association is warranted, especially in Blacks. Our study also underscores the importance of consortium-based replication of genetic epidemiological studies to achieve large sample sizes.

## Figures and Tables

**Table 1:** Characteristics of the OCAC case–control studies included in this report

**Study (Ref)^a^**	**Location**	**Years conducted**	**Control ascertainment**	**Case ascertainment**	**No. of controls/cases**	**SNPs genotyped**
ACS	Australia	2002–2005	Randomly selected from Commonwealth electoral roll. Frequency matched for age and geographical region.	Cancer registries of New South Wales and Victoria.	364/166	rs2740574 (CYP3A4), rs1805386 (LIG4)
AOCS (Beesley *et al*, 2007)	Australia	2002–2006	Randomly selected from Commonwealth electoral roll. Frequency matched for age & geographical region.	Recruited through surgical treatment centres throughout Australia and cancer registries of Queensland, South Australia and West Australia.	698/558	rs2740574 CYP3A4), rs1805386 (LIG4), rs3218536 (XRCC2), rs861539 (XRCC3)
DOVE	Washington, USA	2002–2005	Random-digit dial identification from study area. Frequency matched to cases for race/ethnicity and 5-year age group.	Cases diagnosed with primary invasive ovarian cancer between 2002–2005 from a 13-county area of western Washington state.	744/548	rs3218536 (XRCC2)
GEOCS (Auranen *et al*, 2005)	Northern California, USA	1997–2002	Random-digit dial identification from study area. Frequency matched to cases for 5-year age group and race/ethnicity.	Greater Bay Area Cancer Registry, San Francisco.	419/317	rs1805386 (LIG4), rs3218536 (XRCC2), rs861539 (XRCC3)
HAWAII (Goodman *et al*, 2001a, 2001b)	Hawaii, USA	1993 onwards	Randomly selected from Hawaii Department of Health Annual Survey of the representatives households.	Rapid case ascertainment through Hawaii Tumour Registry.	160/70	rs4680 (COMT), rs4646903 (CYP1A1), rs1048943 (CYP1A1), rs1056836 (CYP1B1), rs2740574 (CYP3A4), rs743572 (CYP17), rs3020450 (ESR2), rs3218536 (XRCC2)
HOPE	NY, OH and PA, USA	2003 onwards	Identified in same regions. Frequency matched for age and ethnicity.	Physician offices, cancer registries and pathology databases from counties of western PA, eastern OH and western NY.	662/297	RS3218536 (XRCC2)
MALOVA (Auranen *et al*, 2005)	Denmark	1994–1999	Random sample of general female population (35–79 years of age) in study area. Selected using computerised Central Population Register and matched to cases for age and geographical region.	Incident cases (35–79 years of age) from municipalities of Copenhagen and Frederiksberg and surrounding counties.	1197/42	rs2740574 (CYP3A4), rs1805386 (LIG4), rs3218536 (XRCC2), rs861539 (XRCC3)
MAYO (Sellers *et al*, 2005)	Mayo Clinic, USA	2000 onwards	Healthy women seeking general medical examination identified through Mayo Clinic. Frequency matched to cases for age, race, and state of residence.	Cases attending Mayo Clinic identified in a six-state surrounding region.	442/325	rs4680 (COMT), rs1048943 (CYP1A1), rs1056836 (CYP1B1), rs3218536 (XRCC2)
NCOCS	North Carolina, USA	1999 onwards	Controls identified from same region. Frequency matched to cases for age and race.	Identified from 48 counties within the region by rapid-case ascertainment.	941/702	rs2740574 (CYP3A4), rs743572 (CYP17), rs3020450 (ESR2), rs1805386(LIG4), rs3218536(XRCC2), rs861539 (XRCC3)
NECC (Garner *et al*, 2002; Terry *et al*, 2003)	New England, USA	1992–2003	Controls identified through random digit dialing, townbooks, and drivers’ license lists. Frequency matched to cases on age and state of residence.	Identified through hospital tumour boards and state cancer registries in New Hampshire and Massachusetts.	484/268	rs4646903 (CYP1A1), rs1048943 (CYP1A1), rs743572 (CYP17), rs3020450 (ESR2)
OVCARE (Holt *et al*, 2007)	Washington, USA	1994–1998	Controls identified through random digit dialing in same three geographic regions as cases.	Incident cases (35–54 years of age) identified by SEER population-based cancer registries serving metropolitan Atlanta, Detroit, and Seattle areas.	577/188	rs4680 (COMT), rs4646903 (CYP1A1), rs1048943 (CYP1A1), rs1056836 (CYP1B1)
SEARCH (Auranen *et al*, 2005)	UK	1991 onwards	Selected from the EPIC-Norfolk cohort of 25,000 individuals aged 45–74, based in the same geographical regions as cases.	Caese<70 years from East Anglian, West Midlands & Trent regions of England. Prevalent cases diagnosed 1991–1998; incident cases diagnosed 1998 onwards.	1221/851	rs1805386 (LIG4), rs3218536 (XRCC2), rs861539 (XRCC3)
SWH (Spurdle *et al*, 2002; Webb *et al*, 2005)	Australia	1985–1996 (cases) 1992 1993 (controls)	From national twin study (one member of pairs of monozygotic twins).	Cancer registries of New South Wales and Queensland, Australia.	275/377	rs2740574 (CYP3A4), rs743572 (CYP17), rs3218536 (XRCC2), rs861539 (XRCC3)
UCI	Orange and San Deigo counties, USA	1994–2004	Controls identified through random digit dialing. Frequency matched to cases by age group and race/ethnicity.	Recruitment of cases was done by rapid case ascertainment through the Cancer Surveillance Program of Orange and San Diego Counties that resides at UCI and is part of the California Cancer Registry.	438/298	rs3218536 (XRCC2)
UKOPS	UK	2006 onwards	Postmenopausal women from the general population participating in the United Kingdom Collaborative Trial of Ovarian Cancer Screening (UKCTOCS). All women followed up for cancers through the Office of National Statistics	Incident cases from ten gynaecological oncology National Health Service centres throughout the UK, from January 2006 onwards.	584/262	rs2740574 (CYP3A4), rs3218536 (XRCC2)
USC (Pearce *et al*, 2008b)	Los Angeles, USA	1993 onwards	Neighborhood recruited controls, frequency matched to cases for age and ethnicity.	Rapid case ascertainment through Los Angeles Cancer Surveillance Program.	701/564	rs743572 (CYP17), rs3020450 (ESR2), rs1805386 (LIG4), rs3218536 (XRCC2)

a See Materials and Methods for full study name.

**Table 2:** Summary odds ratio (per allele) and 95% confidence interval for risk of invasive epithelial ovarian cancer among the indicated OCAC studies for SNPs that showed no association with ovarian cancer

**Gene**	**SNP**	**Controls (*N*)**	**Cases (*N*)**	**OR_het_^a^ (95% CI)**	***P*-value**	**OR_hom_^a^ (95% CI)**	***P*-value**	**Studies**
COMT	rs4680	874	381	1.01 (0.75–1.36)	0.96	1.00 (071–1.42)	0.98	HAWAII, MAYO OVCARE
CYP1A1	rs4646903	1182	490	1.15 (0.89–1.50)	0.29	0.77 (0.28–2.13)	0.61	HAWAII, NECC, OVCARE
CYP1A1	rs1048943	1308	611	1.13 (0.79–1.63)	0.50	—	0.97	HAWAII, MAYO, NECC, OVCARE
CYP1B1	rs1056836	875	384	0.91 (0.69–1.21)	0.52	0.83 (0.58–1.19)	0.31	HAWAII, MAYO OVCARE
CYP17	rs743572	1594	1078	1.09 (0.91–1.30)	0.36	1.19 (0.93–1.52)	0.17	HAWAII, NCOCS, NECC, SWH, USC
ESR2	rs3020450	2198	1523	0.95 (0.83–1.10)	0.52	1.09 (0.87–1.36)	0.44	HAWAII, NCOCS, NECC, USC
XRCC3	rs861539	5186	2352	0.92 (0.82–1.04)	0.17	1.01 (0.85–1.19)	0.95	AOCS, MALOVA, NCOCS, SEARCH, GEOCS, SWH

a CI=confidence interval; het=heterozygous carriers; hom=homozygous carriers; OR=odds ratio. All ORs stratified on study site, race/ethnicity and age.

**Table 3:** Summary odds ratio and 95% confidence interval for risk of invasive epithelial ovarian cancer among OCAC studies for the three SNPs associated with risk of ovarian cancer in original data

				**95% CI**	***P*-value**			
	**Controls (*N*)**	**Cases (*N*)**	**OR_het_^a^**	**CYP3A4 rs2740574^b^**		**OR_hom_^a^**	**95% CI**	***P*-value**
Original data	664	563	1.51	0.85–2.40	0.084	3.66	1.29–10.40	0.015
Replication data	2827	1406	0.96	0.73–1.25	0.75	2.50	0.54–11.54	0.24
Combined data	3491	1969	1.07	0.85–1.34	0.57	2.81	1.20–6.56	0.017
				**LIG4 rs1805386^c^**				
Original data	2196	1630	1.21	1.04–1.40	0.013	1.34	0.90–1.99	0.15
Replication data	2944	1691	0.87	0.75–1.00	0.046	0.67	0.45–1.01	0.056
Combined data	5140	3321	1.01	0.91–1.12	0.82	0.94	0.71–1.25	0.68
				**XRCC2 rs3218536^d^**				
Original data	3668	2763	0.92	0.79–1.06	0.24	0.60	0.31–1.17	0.13
Replication data	4005	2551	1.05	0.91–1.21	0.50	0.79	0.40–1.57	0.50
Combined data	7673	5314	0.97	0.88–1.08	0.61	0.68	0.42–1.09	0.11

a het=heterozygous carriers; OR=odds ratio; hom=homozygous carriers.

b Studies included in original data: HAWAII, NCOCS, SWH; studies included in follow-up data: ACS, AOCS, MALOVA, UKOPS.

c Studies included in original data: GEOCS, NCOCS, SEARCH; studies included in follow-up data: ACS, AOCS, MALOVA, USC.

d Studies included in original data: AOCS, GEOCS, MALOVA, NCOCS, SEARCH, SWH; studies included in follow-up data DOVE, HAWAII, HOPE, MAYO, SEARCH (additional accrued cases), UCI, UKOPS, USC.

**Table 4:** Odds ratios and 95% CIs by race/ethnicity and histological sub-type^a^ for the CYP3A4 rs2740574 variant in the combined dataset

**Characteristics**	**Cases**	**Controls**	**OR_het_^b^**	**95% CI**	***P*-value**	**OR_hom_^b^**	**95% CI**	***P*-value**
Black	24	36	2.86	0.28–29.63	0.38	6.82	0.73–63.86	0.093
White	1944	3453	1.06	0.84–1.33	0.62	2.4	0.67–8.68	0.18
Endometrioid	229	3491	1.65	1.05–2.59	0.03	2.64	0.36–19.16	0.34
Mucinous	107	3491	0.54	0.19–1.51	0.24	4.75	0.47–47.84	0.19
Serous	1052	3491	0.93	0.70–1.24	0.63	2.8	1.00–7.83	0.050

a Clear cell histology not informative.

b OR=odds ratio; het=heterozygous OR; hom=homozygous OR. Studies included: ACS, AOCS, HAW, MALOVA, NCO, SWH, UKOPS; all ORs stratified on study site, age and race/ethnicity (for histology analyses).

## Appendix A

ACS/AOCS: The AOCS gratefully acknowledges the cooperation of the New South Wales, Queensland, South Australian, Victorian and Western Australian Cancer Registries as well as all the collaborating institutions represented within the AOCS Study Group which comprises: Management Group: D Bowtell (Peter MacCallum Cancer Centre, PMCC), G Chenevix-Trench, A Green, P Webb (Queensland Institute of Medical Research, QIMR), A deFazio (Westmead Hospital), D Gertig (University of Melbourne). Project Managers: N Traficante (PMCC), S Moore (QIMR), J Hung (Westmead Hospital). Data Managers: S Fereday (PMCC), K Harrap, T Sadkowsky (QIMR). Research Nurses: NSW – A Mellon, R Robertson (John Hunter Hospital), T Vanden Bergh (Royal Hospital for Women), J Maidens (Royal North Shore Hospital), K Nattress (Royal Prince Alfred Hospital), YE Chiew, A Stenlake, H Sullivan, (Westmead Hospital); QLD – B Alexander, P Ashover, S Brown, T Corrish, L Green, L Jackman, K Martin, B Ranieri (QIMR); SA- J White (QIMR); TAS- V Jayde (Royal Hobart Hospital); VIC – L Bowes (PMCC), P Mamers (Monash Medical Centre), WA – T Schmidt, H Shirley, S Viduka, H Tran, S Bilic, L Glavinas (Western Australia Research Tissue Network, WARTN). Clinical Collaborators: NSW – A Proietto, S Braye, G Otton (John Hunter Hospital); T Bonaventura, J Stewart (Newcastle Mater Misericordiae); M Friedlander (Prince of Wales Hospital); D Bell, S Baron-Hay, A Ferrier, G Gard, D Nevell, B Young (until mid 2003) (Royal North Shore Hospital); C Camaris, R Crouch, L Edwards, N Hacker, D Marsden, G Robertson (Royal Hospital for Women); P Beale, J Beith, J Carter, C Dalrymple, A Hamilton, R Houghton, P Russell (Royal Prince Alfred Hospital); A Brand, R Jaworski, P Harnett, G Wain (Westmead Hospital); QLD – A Crandon, M Cummings, K Horwood. A Obermair, D Wyld (Royal Brisbane and Women's Hospital, RBWH); J Nicklin (RBWH and Wesley Hospital), D Papadimos (SN Pathology), L Perrin (RBWH and Mater Misericordiae Hospitals), B Ward (Mater Misericordiae Hospitals); SA – M Davy, C Hall, T Dodd, T Healy, K Pittman (Royal Adelaide Hospital, Burnside Memorial Hospital); D Henderson, S Hyde (Flinders Medical Centre); J Miller, J Pierdes (Queen Elizabeth Hospital) TAS – P Blomfield, D Challis, R McIntosh, A Parker (Royal Hobart Hospital); VIC- B Brown, R Rome (Freemasons Hospital); D Allen, P Grant, S Hyde, R Laurie M Robbie, (Mercy Hospital for Women), D Healy, T Jobling, T Maniolitas, J McNealage, P Rogers, B Susil, A Veitch, J Constable, S Ping Tong, I Robinson, I Simpson, (Monash Medical Centre); K Phillips, D Rischin, P Waring, M Loughrey, N O’Callaghan, Bill Murray (PMCC); V Billson, S Galloway, J Pyman, M Quinn (Royal Women's Hospital); WA – I Hammond, A McCartney, Y Leung (King Edward Memorial Hospital, St John of God). Scientific Collaborators: I Haviv (PMCC); D Purdie, D Whiteman (QIMR); N Zeps (WARTN). GCT, ABS and PW are supported by the NHMRC. Additional support was provided by: US Army Medical Research and Materiel Command under DAMD17-01-1-0729, the Cancer Council Tasmania and Cancer Foundation of Western Australia (AOCS study); The National Health and Medical Research Council of Australia (199600) (ACS study).

GEOCS: This work was funded by grants from the Roswell Park Alliance and National Institutes of Health Grants CA66190 and CA16056.

Hawaii: US Public Health Service grant R01-CA-58598, and contracts N01-CN-55424 and N01-PC-35137 from the National Cancer Institute, NIH, Department of Health and Human Services.

HOPE: This work was funded by National Institutes of Health Grant R01CA095023 and DAMD 17-02-1-0669.

MAYO: This work was funded by National Institutes of Health Grants R01-CA86888 and R01-CA122443.

NECC: This work was funded by National Institutes of Health Grants 5P50CA105009 and 5RO1CA054419.

NCOCS: This work was funded by National Institutes of Health Grant 2-R01-CA76016.

SEARCH: This work was supported by Cancer Research-UK. We thank: Hannah Munday, Barbara Perkins, Mitul Shah, Clare Jordan, Judy West, Anabel Simpson, Sue Irvine, the search team: the local general practices and nurses and the East Anglian Cancer Registry for recruitment of the UK cases and the EPIC-Norfolk investigators for recruitment of the UK controls; PDPP is CRUK Senior Clinical Research Fellow.

UCI: This research was supported by the National Institutes of Health Grants CA-58860, CA-92044 and the Lon V Smith Foundation Grant LVS-39420.

UKOPS: We thank all members of the research team, including research nurses, research scientists, data entry personnel and consultant gynaecological oncologists for their help in establishing the UKOPS case–control collection. In particular, we thank Eva Wozniak for the efforts in genotyping and data analysis and Andy Ryan and Jeremy Ford for data and sample management.

USC: This work was funded by the California Cancer Research Program Grants 00-01389V-20170 and 2110200, US Public Health Service Grants CA14089, CA17054, CA61132, CA63464, N01-PC-67010 and R03-CA113148, and California Department of Health Services subcontract 050-E8709 as part of its statewide cancer reporting programme.
