# Validating genetic risk associations for ovarian cancer through the International Ovarian Cancer Association Consortium

**DOI:** 10.1038/sj.bjc.6605431

**Published:** 2009-11-10

**Authors:** C L Pearce, A M Near, D J Van Den Berg, S J Ramus, A Gentry-Maharaj, U Menon, S A Gayther, A R Anderson, C K Edlund, A H Wu, X Chen, J Beesley, P M Webb, S K Holt, C Chen, J A Doherty, M A Rossing, A S Whittemore, V McGuire, R A DiCioccio, M T Goodman, G Lurie, M E Carney, L R Wilkens, R B Ness, K B Moysich, R Edwards, E Jennison, S K Kjaer, E Hogdall, C K Hogdall, E L Goode, T A Sellers, R A Vierkant, J M Cunningham, J M Schildkraut, A Berchuck, P G Moorman, E S Iversen, D W Cramer, K L Terry, A F Vitonis, L Titus-Ernstoff, H Song, P D P Pharoah, A B Spurdle, H Anton-Culver, A Ziogas, W Brewster, V Galitovskiy, G Chenevix-Trench, Australian Cancer Study (Ovarian Cancer) Australian Ovarian Cancer Study Group on behalf of the Ovarian Cancer Association Consortium

**Correction to:** *British Journal of Cancer* (2009) **100**, 412–420. doi:10.1038/sj.bjc.6604820 www.bjcancer.com

Upon publication of this paper in Volume 100, one of the co-author's names was incorrectly stated – Julie Cunningham's middle initial was stated as ‘C’ rather than ‘M’. The authors and publishers apologise for this mistake. The correct author list is now shown, above.

