# Supplementary material for: Validating genetic risk associations for ovarian cancer through the international Ovarian Cancer Association Consortium
Source: Br J Cancer. 2009 Jan 6;100(2):412–20. doi: 10.1038/sj.bjc.6604820 (PMC2634713; doi:10.1038/sj.bjc.6604820)
Supplement: Supplementary file 1 [file 6604820x1.doc]

Supplementary Table 1. Minor allele frequency (MAF) and Hardy-Weinberg Equillibrium p-value for White controls by study for each SNP.

	rs4680 (COMT)		rs4646903		rs1048943		rs1056836		rs2740574		rs743572 (CYP17)		rs3020450 (ESR2)		rs1805386 (LIG4)		rs3218536		rs861539 (XRCC3)	
STUDY*	MAF	p-value	MAF	p-value	MAF	p-value	MAF	p-value	MAF	p-value	MAF	p-value	MAF	p-value	MAF	p-value	MAF	p-value	MAF	p-value
ACS									0.04	0.47					0.18	0.81				
AOCS									0.04	0.33					0.17	0.80	0.08	0.40	0.38	0.60
DOVE																	0.08	0.42		
GEOCS															0.16	0.77	0.10	0.51	0.37	0.10
HAWAII	0.47	0.77	0.11	0.29	0.02	0.81	0.38	0.20	0.03	0.04	0.45	0.76	0.32	0.42			0.09	0.88		
HOPE																	0.08	0.19		
MALOVA									0.04	0.78					0.17	0.69	0.07	0.83	0.38	0.10
MAYO	0.47	0.38			0.05	0.35	0.41	0.42									0.08	0.91		
NCOCS									0.04	0.58	0.39	0.46	0.32	0.50	0.18	0.63	0.09	0.91	0.35	1.00
NECC			0.11	0.60	0.04	0.39							0.33	0.61						
OVCARE	0.46	0.19	0.12	0.95	0.04	0.40	0.44	0.65												
SEARCH															0.15	0.56	0.09	0.48	0.37	0.21
SWH									0.03	0.12	0.38	0.48					0.09	0.37	0.37	0.82
UCI																	0.07	0.65		
UKOPS									0.03	0.48							0.10	0.78		
USC											0.37	0.65	0.33	0.60	0.17	0.52	0.08	0.35		

*See methods for full study name
